# Supplementary material for: Empirical methods for controlling false positives and estimating confidence in ChIP-Seq peaks
Source: BMC Bioinformatics. 2008 Dec 5;9:523. doi: 10.1186/1471-2105-9-523 (PMC2628906; doi:10.1186/1471-2105-9-523)
Supplement: Additional File 1 — A variety of html documents from the USeq web site detailing the available applications, their best usage, output file type descriptions, command line menus, etc. [file 1471-2105-9-523-S1.zip › USeqUserGuides/JavaDoc.zip!/JavaDoc/bioroot/class-use/SearchHit.html]

Uses of Class bioroot.SearchHit


|  |  |  |
| --- | --- | --- |
| **Overview** \| **Package** \| **Class** \| **Use** \| **Tree** \| **Deprecated** \| **Index** \| **Help** | |  |
| PREV   NEXT | **FRAMES**    **NO FRAMES**     **All Classes** |  |


---


## **Uses of Class bioroot.SearchHit**

| Packages that use SearchHit | |
| --- | --- |
| **bioroot** |  |

Uses of SearchHit in bioroot

| Methods in bioroot that return SearchHit | |
| --- | --- |
| `SearchHit[]` | `SearchBean.searchForSimilarMatches(java.lang.String[] columns, java.lang.String[] words, DBUtil util, boolean searchForWordBoundaryMatches, UserBean userBean)`             Searches a list of columns for a set of words, case insensitive, %word% matches. |

---


|  |  |  |
| --- | --- | --- |
| **Overview** \| **Package** \| **Class** \| **Use** \| **Tree** \| **Deprecated** \| **Index** \| **Help** | |  |
| PREV   NEXT | **FRAMES**    **NO FRAMES**     **All Classes** |  |


---
